# Reconstruction of the pulmonary posterior wall using in situ autologous tissue for the treatment of pulmonary atresia with ventricular septal defect

**DOI:** 10.1186/s13019-017-0578-4

**Published:** 2017-02-23

**Authors:** Chengming Fan, Yifeng Yang, Lian Xiong, Ni Yin, Qin Wu, Mi Tang, Jinfu Yang

**Affiliations:** Department of the cardiovascular surgery, the Second Xiangya Hospital, Central South University, Middle Renmin Road 139, 410011 Changsha, China

**Keywords:** Congenital heart disease, Pulmonary atresia, Pulmonary reconstruction, In situ autogenous tissue

## Abstract

**Background:**

To evaluate the early and mid-term results of pulmonary trunk reconstruction using a technique in which autogenous tissue is preserved in situ in pulmonary atresia patients with a ventricular septal defect (PA-VSD).

**Methods:**

The pulmonary artery was reconstructed using autogenous tissue that had been preserved in situ and a bovine jugular venous patch in 24 patients who were diagnosed with PA-VSD (the observation group). The traditional operation using a bovine jugular venous conduit was performed in 40 other cases of PA-VSD (the control group).

**Results:**

In the observation group, all patients survived and recovered successfully without complications. Follow-up echocardiography 2–10 years after the procedure showed that the reconstructed right ventricular outflow tract (RVOT) and pulmonary artery were patent, showing no evidence of flow obstruction. Only mild regurgitation of the bovine jugular vein valve was observed. In the control group, early postoperative death occurred in two cases. Another two patients had obstruction of the anastomotic stoma and underwent conduit replacement surgery within 2 weeks of the initial procedure. During the 2–10 years of follow-up care, six patients presented with valvular stenosis of the BJVC, with a pressure gradient of more than 50 mmHg.

**Conclusions:**

The technique for preserving autogenous tissue to reconstruct the pulmonary posterior wall is a satisfactory method for treating PA-VSD.

## Background

Pulmonary atresia with ventricular septal defect (PA-VSD) is a rare and complex cyanotic congenital heart malformation that has a high incidence of early mortality. Although surgical interventions have been employed successfully for a number of years, the long-term prognosis has remained poor with an increased incidence of sudden death [1]. In 1955, Lillehei et al. successfully corrected an atresia of the main pulmonary artery (MPA) and a ventricular defect in a patient who had been diagnosed with PA-VSD by anastomosing the distal pulmonary artery with the right ventricle [2]. A decade later, Rastelli and colleagues reported a case in which a cardiac conduit from the right ventricle to the pulmonary trunk was used to treat the abnormality [3]. Various extracardiac valved conduits have subsequently been used to correct a right ventricle to pulmonary artery discontinuity since Ross and Somerville successfully employed a homograft aortic valved conduit in 1966 [4]. Weldon et al. reported the same result 2 years later [5]. In 1973, PA-VSD was successfully employed by Bowman and colleagues, who used a Dacron conduit containing a porcine heterograft aortic valve [6]. Despite these improvisations to the Rastelli procedure, most patients with PA-VSD have had to undergo repeated surgeries resulting from first conduit failure because of either calcification or degeneration of the extracardiac graft or a mismatch of biologic prosthesis. Thus, treating a ventricle-pulmonary artery discontinuity remains a major challenge; surgeons have tried to develop an ideal prosthesis while concomitantly developing techniques to reconstruct the right ventricular outflow tract (RVOT) [7, 8]. Herein, we describe a strategy that was performed on 24 patients who were diagnosed with PA-VSD in which autogenous tissue was preserved in situ as the posterior part of a newly reconstructed pulmonary artery. Forty PA-VSD patients underwent control surgeries using the Rastelli procedure of connecting a bovine jugular venous conduit (BJVC) between the right ventricle and the bifurcation of the MPA.

## Methods

### Clinical data

**Fig. 1:**
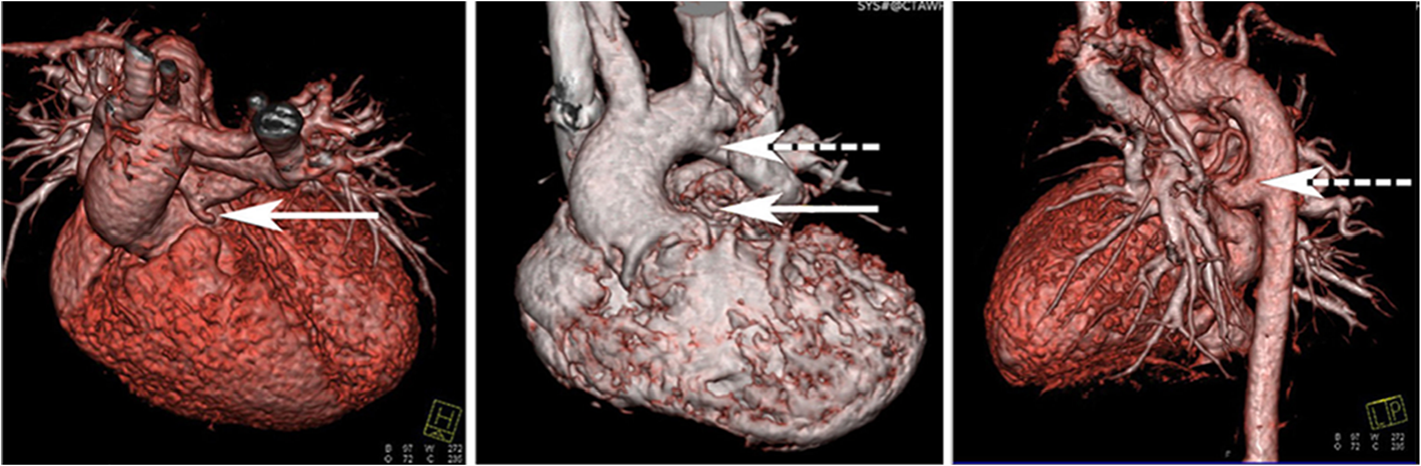
Computed tomography angiography pre-surgery. *Block arrows highlight* the pulmonary atresia, and the *dashed arrows highlight* the patent ductus arteriosus (PDA) or the major aortopulmonary collateral arteries (MAPCA)

At the Second Xiangya Hospital of Central South University from January 2007 to December 2015, 24 children who had been diagnosed with PA-VSD underwent a pulmonary artery reconstruction technique in which autogenous tissue was preserved in situ. In the control group of 40 other PA-VSD patients, the Rastelli procedure was conducted, in which a BJVC was placed from the right ventricle to the pulmonary artery confluence (Table 1). None of the patients had previously received surgical intervention, and patients with MAPCAs that required unifocalization were eliminated from the study. Severe cyanosis was typically observed in all cases, and the level of skin oxygen saturation in the limbs ranged from 65 to 82% at rest conditions. Chest X-rays showed a typical appearance of a “boot-shaped” heart. The diagnosis of PA-VSD was confirmed by echocardiography and computed tomography (CT) cardiac imaging. Echocardiography demonstrated hypertrophy of the right atrium and ventricle, no pulmonary blood flow directly from the right ventricle to pulmonary artery, and a ventricular septal defect (VSD). Additionally, 44 cases had patent ductus arteriosus (PDA), and 36 cases had major aortopulmonary collateral arteries (MAPCA) of the descending aorta, with 16 cases having both PDA and MAPCA. Examination by CT cardiac imaging showed neither a direct connection between the right ventricle and the pulmonary artery nor that PDA or MAPCA co-existed (Fig. 1). The blood supply of the lung was accurately assessed by multidetector computed tomography angiography and/or selective catheter angiography to confirm that pulmonary artery confluence existed and that no parts of the lung were supplied only by the MAPCAs. There were no major coronary arteries crossing the RVOT in any patients. Prior consent from all patients and approval from the local Institutional Research Ethics Committee were obtained.

**Table 1:** Preoperative data from the two groups

	Observation group	Control group	*P* Value
Gender (male/female)	10/14	16/24	0.311
Age (month median)	12.12	19.84	0.001
Body weight (kg median)	9.478	12.870	0.003
Hemoglobin (g/L)	162 ± 19	165 ± 13	0.721
Oxygen blood pressure (mmHg)	71 ± 9	69 ± 7	0.589
Pulmonary artery index (mm^2^/m^2^)	175 ± 15	179 ± 11	0.513
Mcgoon index (median)	1.72	1.76	0.483
PA-VSD Type A (case)	18	30	
PA-VSD Type B (case)	6	10

### Surgical procedure

**Fig. 2:**
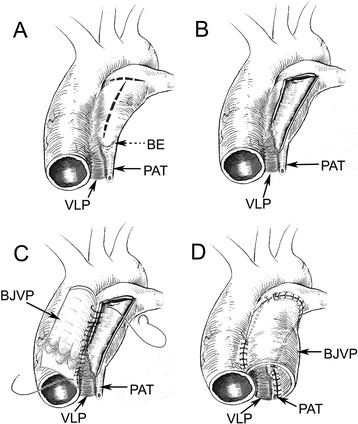
Diagram of the procedure for pulmonary atresia. **a** Thorough examination of PAT, VLP and the BE of pulmonary. **b** Longitudinal incisions of the inherent pulmonary artery to the BE of pulmonary with the reservation of the PAT and VLP. **c** A suitable BJVP was selected and then continuously sutured with left lateral wall of the ascending aorta. **d** Continuously sutured the other end of BJVP with the PAT to reconstruct a new pulmonary artery whereby PAT and VLP were preserved for the partial pulmonary right lateral and posterior wall. Figure **a**–**d**: BE: blind end of pulmonary artery; BJVP: bovine jugular venous patch; PAT: pulmonary atresic tissue; VLP: visceral layer of pericardium

All patients underwent a cardiopulmonary bypass surgical procedure under moderate hypothermia (28–30 °C). Following a thorough examination of the left and right branches of the distal inherent pulmonary artery, the blind end inherent pulmonary artery (BE), visceral layer of pericardium (VLP) and the long fibrous cord of pulmonary atresic tissue (PAT) (Fig. 2a), a right ventricular longitudinal incision was made and extended. Similarly, the anterior wall of the inherent pulmonary artery was longitudinally incised to the BE (Fig. 2b), with the PAT and VLP being reserved. A suitable BJVC (diameter from 14 mm to 18 mm (mean 15 mm) according to the body weight and the visceral pericardium and atresic pulmonary artery tissue) was selected and longitudinally incised along the junction of the valve leaflets to prepare a bovine jugular venous patch (BJVP). The BJVP was then continuously sutured with the left lateral wall of the ascending aorta (Fig. 2c) and the PAT to reconstruct a new pulmonary artery (mean diameter is 17 mm), from which autologous tissue (the PAT, the VLP and the left lateral wall of the ascending aorta) was preserved for the partial pulmonary right lateral and posterior wall (Fig. 2d). While suturing the brim of the longitudinal incision and the margin of the BJVP, it was observed that the bovine jugular venous valves should be sutured in situ with the normal pulmonary valves without injuring the nearby coronary artery. In the control group, a BJVC (diameter from 15 mm to 19 mm (mean 17 mm) according to the body weight) between the right ventricle and pulmonary artery confluence was routinely constructed. All of the children in this study received anticoagulation therapy with low-dose aspirin for 6 months post-surgery.

### Statistical analysis

Data are expressed as the mean ± SE and median. All statistical calculations were performed using the Statistical Product and Service Solutions 14.0 software (SPSS Institute). *X* ^2^ test, Log-rank test and Gehan-Breslow-Wilcoxon test were performed to determine differences between the two groups. The critical alpha level for these analyses was set at *p* < 0.05.

## Results

**Fig. 3:**
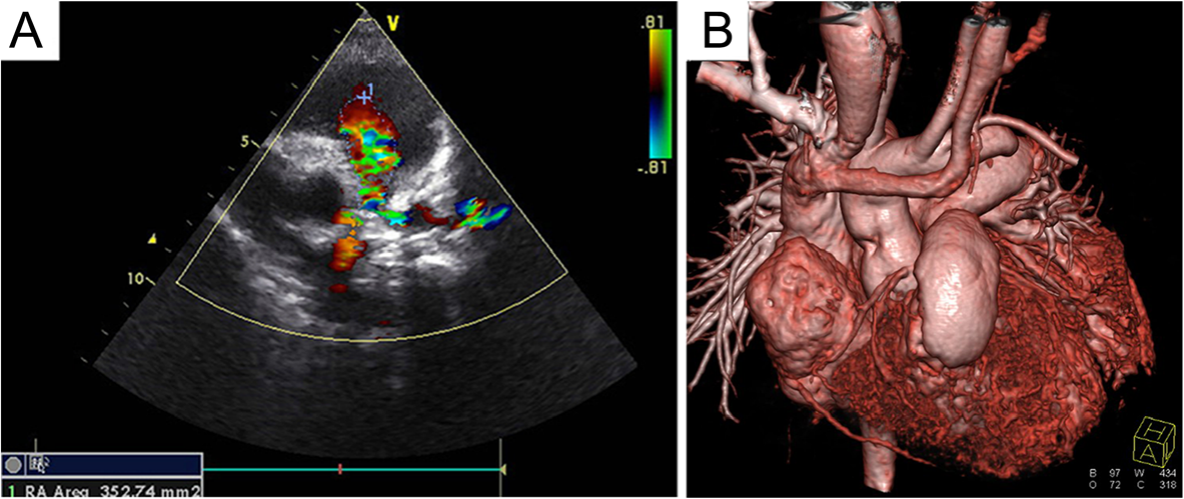
Echocardiography and computed tomography angiography post-surgery. **a** The echocardiographic findings of a case 6 months after the operation shows the blood passing through the reconstructed right ventricular outflow tract (RVOT) and pulmonary artery with a slightly higher speed and only mild pulmonary regurgitation; **b** Postoperative computed tomography angiography shows that the reconstructed RVOT and pulmonary trunk consist of the bovine jugular venous patch (BJVP) and the autologous tissue

**Fig. 4:**
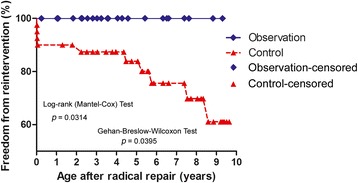
Kaplan-Meier curves. Freedom from re-intervention during the follow-up period of the two groups was significant differences

The mean bypass and cross clamp times were similar in the observation and control groups. The postoperative ventilator support time, the duration of stay in the intensive care unit (ICU), and postoperative residence time were also not significantly different between the groups (Table 2). Postoperative examination in both groups showed complete repair of the cardiac malformations. There were no manifestations of cyanosis and the level of skin oxygen saturation in the limbs rose to 96–100% at rest conditions. In the observation group, follow-up echocardiography 2–10 years post-procedure (mean follow-up duration was 4.196 years) showed that the reconstructed RVOT and pulmonary artery were patent, with no evidence of flow obstruction or residual VSD. Only mild regurgitation of the bovine jugular vein valve was observed in all patients (Fig. 3a). CT cardiac imaging also confirmed that the morphology and function of the reconstructed RVOT and pulmonary artery appeared appropriate in all cases (Fig. 3b). In the control group, two patients had low cardiac output syndrome and anuria that showed short-term improvement during peritoneal dialysis treatment; however, after 12 and 20 days, they died of multiple organ failure and disseminated intravascular coagulation. Another two cases had anastomotic obstruction and underwent conduit replacement surgery within 2 weeks of the initial procedures because of pulmonary anastomotic stenosis (pressure gradient of 46 mmHg) and obstruction angulation of conduit (pressure gradient of 32 mmHg). Further, during the 2–10 years of follow-up care (mean follow-up duration is 5.378 years) in the control group, 36 patients presented with varying degrees of valvular stenosis of the BJVC. As a result, 4 patients with a pressure gradient greater than 50 mmHg underwent balloon valvuloplasty due to poor valve activity, and two patients with a pressure gradient greater than 85 mmHg underwent conduit replacement. Statistical analysis showed significant differences of freedom reintervention between these groups (Fig. 4).

**Table 2:** Postoperative data from the two groups

	Observation group	Control group	*P* Value
Cardiopulmonary bypass time (min)	102 ± 18	99 ± 22	0.733
Aortic cross clamp time (min)	85 ± 10	82 ± 13	0.409
Mechanical ventilation time (h)	68 ± 23	73 ± 18	0.313
ICU residence time (d)	5.5 ± 2.2	6.2 ± 2.4	0.198
Postoperative residence time (d)	20 ± 3	26 ± 2	0.208
Re-intervention (case)	0	8	0.031

## Discussion

PA-VSD accounts for approximately 2% of all congenital heart disease cases and is one of the most common causes of cyanosis and hypoxemia in neonates. The pathological anatomic characteristics of PA-VSD include VSD, intramuscular or valvar atresia of the pulmonary artery, an overriding of the aorta, and PDA or MAPCAs. According to the classification presented by Castaneda et al., there are four types of PA-VSD [9]. In this study, 48 of the patients were type A, and 16 patients were type B (Table 1). Selection of the surgical procedure for treatment of PA-VSD depends mainly on development of the right ventricle and the pulmonary artery [10]. There are two evaluation indices for pulmonary artery development that can be considered: the McGoon index and the pulmonary artery index (PAI) [11]. A patient is considered suitable for radical repair if the McGoon index is greater than 1.2 or the PAI is greater than 150 mm^2^/m^2^, suggesting no severe hypoplasia of the pulmonary artery, which indicates that a patient would be a suitable candidate for radical repair.

Currently, the most common method of radical repair for the treatment of PA-VSD is the classical Rastelli procedure, in which a right ventriculotomy is repaired with an artificial patch. Following the ligation of the co-existing PDA or MAPCAs, the remnant pulmonary trunk is excised, the proximal end is closed, and a vascular prosthesis is then used to connect the right ventricle and the pulmonary artery.

Despite the short-term success of employing different conduit modifications of the Rastelli procedure [7, 8, 12–14], many common complications remain. These include conduit dilation, twisting, thrombosis, calcification, endocarditis, arrhythmia and re-stenosis [15–17]. Shebani et al. [12] found that the early mortality rate resulting from conduit dilation, complete conduit thrombosis, and sudden arrhythmia was 6.4%, as determined from a retrospective analysis of 62 cases of RVOT and pulmonary trunk reconstruction using a bovine Contegra valved conduit. Distal conduit stenosis at the suture line associated with a size-mismatch with the conduit being too small was the main indicator for conduit-related secondary intervention. The high incidence of conduit-related complications, particularly with smaller conduits, included characteristics such as neointimal proliferation, thrombosis, calcification, and chronic inflammation. Reoperation is therefore inevitable for some patients with pulmonary atresia (PA) who receive a heterograft or homograft in a primary Rastelli operation. To treat PA-VSD, Chiu and colleagues [13] adopted a method used in the repair of Fallot’s tetralogy, a congenital heart defect that is understood to involve four anatomical abnormalities, in which a piece of fresh pericardium is harvested and sutured to cover the anterior part of the RVOT. However, pulmonary regurgitation and tricuspid regurgitation are common complications of this procedure. Schreiber et al. [14] replaced the traditional conduit with the Shelhigh No-React (NR-4000PA series)-treated porcine pulmonic valve conduit (SPVC). However, they concluded that a small-sized SPVC cannot be considered an ideal conduit in the reconstruction of the RVOT because although the No-React-treated valve largely resisted calcification, pseudointimal peel formation was found in all explanted conduits and led to multilevel conduit stenosis. Moreover, after reviewing the long-term results of RVOT and pulmonary trunk reconstruction with homografts, Kim et al. [7] determined that although long-term survival was excellent, freedom from reoperation was unsatisfactory, particularly in patients who had small grafts in the initial repair. After analyzing a large patient cohort, Sekarski et al. concluded that smaller pulmonary arteries increase the risk of surgical intervention when using a BJVC to reconstruct the RVOT [8]. Thus, alternative surgical strategies that do not employ small grafts must be considered for young children. In most Chinese areas, especially in rural areas, because of economic considerations, the patient’s custodian tends to refuse to repeat surgical procedures; thus, the BJVC were more commonly used in older children, and BJVP were used more in young children, as shown in Table 1.

**Fig. 5:**
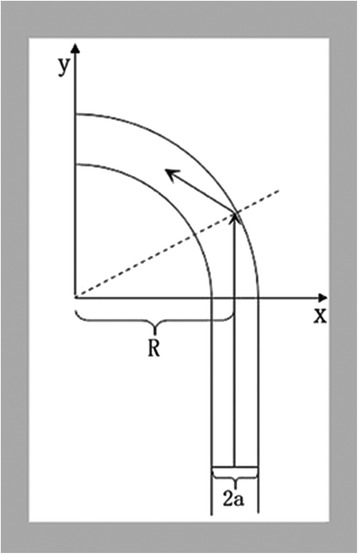
Schematic illustration. Blood flow in a curved conduit illustrating the impact on the wall and energy loss

Another problem with using an extra-cardiac conduit is that it is difficult to achieve a physiologic linear flow. In the 1920s, Dean [18] described the phenomenon of secondary flow when he used a constant and circular cross-section curved vessel model in a study of the streamline motion of fluid in a curved pipe. As a result, scholars later defined $\mathrm{D}\mathrm{e}=4\ \mathrm{R}\mathrm{e}\sqrt{2 a/ R}$ as the Dean number, where Re is the parameter of Reynolds, *a* is the radius of the pipe, and R is the curvature radius of the curved pipe (Fig. 5). The lower the degree of curvature is, the smaller the resulting Dean number is. This implies a smaller impact force against the wall with resultant lower energy losses. When parameter R approaches infinity, the pipe tends to be straight, and the flow tends to be linear. On the one hand, energy consumption of blood flow is partially used for the vascular wall, leading to an additional right ventricular load; on the other hand, the force against the wall brought by deviating blood flow can itself induce the dilation and failure of a vascular prosthesis. Moreover, secondary flow can lead to the accumulation of blood cells, resulting in conduit failure resulting from a build-up of platelets on the outer side of the curve.

To address the problems described, the 24 cases in this study were radically repaired using a procedure in which autogenous tissue was preserved in situ to reconstruct the pulmonary posterior wall. During the follow-up care period, only mild regurgitation of the bovine jugular vein valve was observed in all patients. In the control group, two patients had severe anastomotic stenosis and underwent a further conduit replacement procedure in the early postoperative stage, four patients underwent balloon valvuloplasty and two patients underwent conduit replacement due to stenosis of the BJVC. The most significant differences between the procedures described in the observation group and the control group are as follows: 1. The was a more efficient relief of the right ventricular outflow tract obstruction. 2. The linear bloodstream flow solved the problem of right ventricular overload compared with a traditional conduit that involves the grade climbing of non-linear bloodstream flow. 3. A valved conduit reduced regurgitation, even when there was a gap present between the posterior wall and the bovine valve leaflets. It is important to note, however, that the minimal pulmonary regurgitation did not affect the function of the right ventricle. 4. This technique avoided the severance of remnant pulmonary trunk. Because the preserved autologous vascular bed could stimulate the continual development of the residual pulmonary trunk, young patients, especially children, who receive this procedure will generally not require future conduit replacement. 5. This surgical technique is suitable for PA and does not carry a risk of the major coronary arteries crossing the RVOT.

## Conclusion

Reconstructing the pulmonary posterior wall by using in situ autologous tissue is a satisfactory method for treating PA-VSD. Increasing the sample size and continued follow-up care are necessary to fully assess the long-term benefits and advantages of the procedure.

## Abbreviations

BE: the blind end of inherent pulmonary artery
BJVC: bovine jugular venous conduit
BJVP: bovine jugular venous patch
CPB: cardiopulmonary bypass
CT: computed tomography
MAPCA: major aortopulmonary collateral arteries
PA: Pulmonary atresia
PAI: Pulmonary artery index
PAT: Pulmonary atresic tissue
PA-VSD: Pulmonary atresia with ventricular septal defect
PDA: Patent ductus arteriosus
RVOT: Right ventricular outflow tract
RVOTO: Right ventricular outflow tract obstruction
SPVC: Shelhigh No-React (NR-4000PA series)-treated porcine pulmonic valve conduit
VLP: Visceral layer of pericardium
VSD: Ventricular septal defect
